# Comprehensive Analysis of Prognostic Markers for Acute Myeloid Leukemia Based on Four Metabolic Genes

**DOI:** 10.3389/fonc.2020.578933

**Published:** 2020-09-29

**Authors:** Yuanyuan Zhang, Shengling Ma, Moran Wang, Wei Shi, Yu Hu

**Affiliations:** ^1^Department of Oncology, Tongji Medical College, Tongji Hospital, Huazhong University of Science and Technology, Wuhan, China; ^2^Institute of Hematology, Union Hospital, Tongji Medical College, Huazhong University of Science and Technology, Wuhan, China; ^3^Fred Hutchinson Cancer Research Center, Seattle, WA, United States

**Keywords:** bioinformatic analysis, metabolism, acute myeloid leukemia, survival, prognosis

## Abstract

**Background:** Metabolic reprogramming is the core characteristic of tumors during the development of tumors, and cancer cells can rely on metabolic changes to support their rapid growth. Nevertheless, an overall analysis of metabolic markers in acute myeloid leukemia (AML) is absent and urgently needed.

**Methods:** Within this work, genetic expression, mutation data and clinical data of AML were queried from Genotype-Tissue Expression (GTEx) database, The Cancer Genome Atlas (TCGA) database and Gene Expression Omnibus (GEO) database. The tumor samples of TCGA were randomly divided into a training group (64 samples) and an internal validation group (64 samples) at one time, and the tumor samples of GEO served as two external validation groups (99 samples, 374 samples). According to the expression levels of survival-associated metabolic genes, we divided all TCGA tumor samples into high, medium and low metabolism groups, and evaluated the immune cell activity in the tumor microenvironment of the three metabolism groups by single-sample gene set enrichment analysis (ssGSEA) algorithm. Finally, we examined the mutations and prognostic effects of each model gene.

**Results:** Four metabolism-related genes were screened and applied to construct a prognostic model for AML, giving excellent results. As for the area under the curve (AUC) value of receiver operating characteristic (ROC) curve, the training group was up to 0.902 (1-year), 0.81 (3-year), and 0.877 (5-year); and the internal and external validation groups also met the expected standards, showing high potency in predicting patient outcome. Univariate and multivariate prognostic analyses indicated that the riskScore obtained from our prognostic model was an independent prognostic factor. ssGSEA analysis revealed the high metabolism group had higher immune activity. Single and multiple gene survival analysis validated that each model gene had significant effects on the overall survival of AML patients.

**Conclusions:** In our study, a high-efficiency prognostic prediction model was built and validated for AML patients. The results showed that metabolism-related genes could become potential prognostic biomarkers for AML.

## Introduction

AML is a malignant disease of myeloid hematopoietic stem/progenitor cells, which is mainly characterized by primitive and immature myeloid cell dysplasia in the bone marrow and peripheral blood. Its clinical manifestations are anemia, hemorrhage, infection and fever, organ infiltration and metabolic abnormality, etc. In most cases, the condition is urgent and severe, and the prognosis is poor, which may endanger life if not treated in time (1). Chemotherapy, targeted drugs and hematopoietic stem cell transplantation are still the main approaches for AML treatment. Among them, even the most ideal treatment methods such as intensive chemotherapy and allogeneic hematopoietic stem cell (HSC) transplantation, the cure rate is relatively low (2). Therefore, it is urgent to explore new and accurate biomarkers to evaluate the diagnosis and prognosis of AML patients.

More and more evidences show that the metabolic pattern of cell carcinogenesis has changed significantly, which involves many aspects such as glycolysis, TCA cycle, oxidative phosphorylation, amino acid metabolism, fatty acid metabolism and nucleic acid metabolism (3). This phenomenon is known as the metabolic reprogramming of tumor cells (4). Metabolic reprogramming is one of the important hallmarks of tumors. The rapidly proliferating tumor cells take high rate glycolysis as the primary energy supply method to promote the adaptation to the stress environment such as hypoxia and increase the malignant potential of tumors (5). Thus, metabolic reprogramming can be further used to diagnose, monitor and treat cancer. In recent years, new metabolic inhibitors have been developed for the clinical treatment of cancer (6–9).

Metabolic reprogramming also has a significant impact on the progression, treatment and prognosis of AML (10, 11). More precisely, there are abnormalities in the metabolic processes of AML such as glycolysis, amino acid metabolism, fatty acid metabolism, epigenetic modification and autophagy pathway (12). In addition, metabolic abnormalities may promote the immune escape of AML, resulting in immunotherapy limitations (13). A series of novel drugs targeting the metabolic processes of AML have been developed and studied in preclinical and clinical trials (14–16).

The popular idea is that metabolism regulates the strength of the immune system (17). An important study found that arginine metabolism in AML inhibited T cell proliferation, becoming a potential therapeutic target (18). However, a proteomic analysis showed that lipid metabolism increased the melanoma sensitivity to T-cell-mediated killing by promoting antigen presentation (19). Similarly, in melanoma and breast tumors, the treatment targeting pyruvate metabolic reprogramming increased the immune cells infiltration by reducing the lactic acid production and neutralizing the tumor acidity, and finally played a significant role in inhibiting tumor growth (20). Relevant metabolic reprogramming agents, such as leptin, which provides metabolic support for tumor immunity, are also being developed (21). Thus, for tumor microenvironment (TME), different metabolic pathways have different effects on immune cell adaptability and effector function. Overall, the association between the metabolic state of AML and immune response may be the basis for improving the immunotherapy response in the future, with broad exploration space and application prospects.

We used a variety of bioinformatics analysis methods to explore metabolism-related prognostic factors in AML. COX regression analysis helped us screen out significant prognostic markers for further study. The metabolic prognostic model constructed by the training group showed excellent predictive performance after the double validation and the model gene survival analysis. These results provide a basic direction for further exploration of the molecular mechanism and diagnostic markers of AML.

## Methods

### Data Acquisition and Differential Analysis

GTEx database contains healthy human samples of 42 tissue types, covering almost all the transcriptional genes (22). TCGA database stores a large amount of genomic data and clinical data, which provides a basis for the exploration of meaningful genomic changes and biological mechanisms affecting tumor initiation, development, differentiation and metastasis (23). In order to unify standards, we downloaded the GTEx gene expression dataset (GTEX_RSEM_gene_fpkm), the GTEx sample information file (GTEX_phenotype), the TCGA-LAML gene expression dataset (TCGA-LAML.htseq_fpkm) and the TCGA-LAML sample information file (TCGA-LAML.survival) from the University of California Santa Cruz (UCSC) genome database (24). We also retrieved AML probe matrix files (GSE71014_series_matrix, GSE37642_series_matrix) and platform files (GPL10558-50081, GPL96-57554) from the GEO database (25). Metabolism-related genes were obtained from the Gene Set Enrichment Analysis (GSEA) website (26). In addition, the AML mutation data (TCGA.LAML.varscan.e595f93d-41ac-435e-8c90-06df7e9d6742.DR-10.0.somatic) was also downloaded from TCGA website. All AML samples from the TCGA and GEO databases were bone marrow samples from patients with initial diagnosis.

All AML gene expression data from TCGA and GEO databases had been processed by log2(x+1) and genes with expression values close to 0 were deleted to exclude the influence of extreme values or outliers. Then, we extracted the gene expression data of normal blood samples (337 samples) from the GTEx gene expression dataset, and the gene expression data of AML samples (151 samples) from the TCGA-LAML gene expression dataset. These two gene expression data were merged, using the limma package (27) for standardized processing, and the expression of metabolic genes (945 genes) was extracted simultaneously to facilitate subsequent analysis. We intersected the metabolic genes of GEO and TCGA, correcting the batch effect with the sva package (28). We used the Wilcox test to analyze the difference between the normal group and the tumor group, screening differential metabolic genes (275 genes) according to FDR <0.05 and | logFC | >0.5, and drawing differential heatmap (pheatmap R package) and volcano plot finally.

### Construction of Prognostic Model and Survival Analysis

After combining the expression of differential metabolic genes with survival time, we divided TCGA samples (128 samples) randomly at one time into a training group (64 samples) and an internal validation group (64 samples), with the GEO samples as two external validation groups (99 samples, 374 samples). For the training group, univariate COX analysis (*P* < 0.01) found survival-associated metabolic genes (12 genes), and the least absolute shrinkage and selection operator (LASSO) (29) removed the high correlation genes to prevent the over-fitting of the model by using the R package glmnet (8 genes). Finally, stepwise multivariate Cox regression analysis was performed to construct the optimal prognostic model (4 genes). Meanwhile, in the SPSS 26 version software, we used Schoenfeld residuals to carry out the PH assumption test on model genes, and also drew a plot of the Schoenfeld Residuals against the transformed time for each model gene to ensure the assumptions of proportional hazards is met.

The patient riskScores of the training and validation groups were calculated according to the constructed prognostic model formula. With the median riskScore of the training group as the threshold, we divided the patients in the training and validation groups into a high-risk group and low-risk group, plotting the survival curve (survival R package), ROC curve (survivalROC R package) and risk curve of the training and validation groups, respectively. Finally, univariate and multivariate prognostic analyses were performed for the training group (*P* < 0.05) to judge whether the riskScore obtained from the model could be an independent prognostic factor.

### ssGSEA Analysis and Mutation Data Visualization

We conducted ssGSEA analysis using GSVA package (30) to obtain the immune activity of 29 immune-related genesets in TCGA-AML samples, and the correction results were between 0 and 1. According to the expression of survival-associated metabolic genes (*P* < 0.05), hclust function was applied to cluster TCGA-AML samples, generating high, medium and low metabolism groups. Based on the correlation analysis using ESTIMATE algorithm between metabolism groups and tumor microenvironment, the heat map (pheatmap R package) and violin plots (ggpubr R package) of tumor microenvironment were drawn. The survival curves of the three metabolism groups were plotted after survival analysis. We performed the GSEA enrichment analysis using the org.Hs.eg.db R package between the high and low metabolism groups, drawing the top 5 Gene Ontology (GO) terms and Kyoto Encyclopedia of Genes and Genomes (KEGG) pathways, respectively, that were enriched most significantly in the high metabolism group (*P* < 0.05).

The maftools package (31) helped to visualize the TCGA-AML mutation data, drawing waterfall plots of the high, medium and low metabolism groups, respectively.

### Mutation Status and Multiple Validations of Model Genes

We entered the cBioportal website (32) and selected the study (Acute Myeloid Leukemia TCGA PanCancer data) to download the mutation status of model genes.

To validate the expression differences of model genes, the differences of model genes were analyzed between the high-risk group (69 samples) and the low-risk group (59 samples) of TCGA-AML, with the boxplots of each model gene plotted using the ggpubr package.

In order to validate the prognostic effect of model genes, Gene Expression Profiling Interactive Analysis (GEPIA) website (33) was used to conduct a single gene survival analysis for each model gene firstly, in which Cutoff-High was 70% and Cutoff-Low was 30%; Next, we applied the PROGgeneV2 online tool (34) and selected the TCGA–AML dataset to conduct multiple gene survival analysis for all model genes.

### Statistical Analyses

All statistical analyses were conducted by using R software (version 3.6.3) unless otherwise stated. Univariate and multivariate Cox regression analyses were conducted to investigate the prognostic value of AML-related metabolic signature. All statistical results with a *P* < 0.05 were considered significant.

## Results

### Metabolic Genes Differentially Expressed Between Normal and Tumor Samples

**Figure 1:**
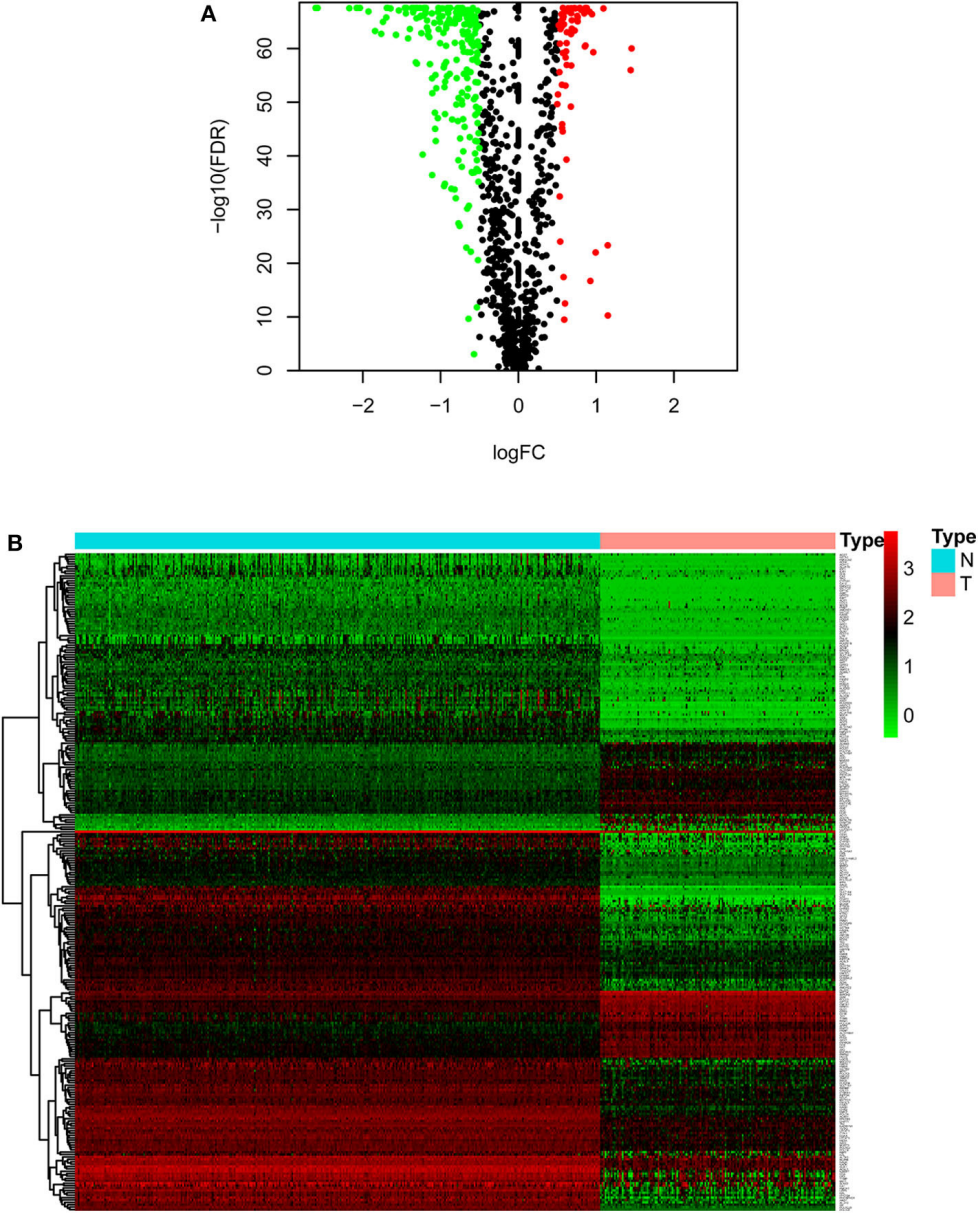
Volcano plot and heatmap of differential metabolic genes. **(A)** Volcano plot. Black dots, green dots and red dots represent undifferentiated genes, down-regulated genes in the tumor group and up-regulated genes in the tumor group, respectively. **(B)** Heatmap. The blue type represents the normal samples and the red type represents the tumor samples. Green color, black color and red color in the main body correspond to low expression, medium expression and high expression.

As described in the methods section, we downloaded AML clinical information (Table 1, Supplementary File 1) and corrected AML gene expression data from the public platforms, and analyzed the differences between normal and tumor samples by Wilcox test, screening 275 differentially expressed metabolic genes by FDR <0.05 and | logFC | >0.5 (Supplementary File 2) and drawing differential heatmap and volcano plot finally. The volcano plot shows the differences distribution in the expression levels of metabolic genes between normal and tumor samples on the whole (Figure 1A). The heatmap depicts expression changes of each metabolic gene between normal and tumor samples (Figure 1B).

**Table 1:** Clinical characteristics of AML patients in the TCGA database.

**Characteristics**		**Total**	**%**
**All**		**171**	**100.00**
Age (y)	≥60	73	42.69
	<60	98	57.31
Gender	Male	92	53.80
	Female	79	46.20
FAB category	M0	14	8.19
	M1	36	21.05
	M2	38	22.22
	M3	18	10.53
	M4	40	23.39
	M5	18	10.53
	M6	3	1.75
	M7	3	1.75
	Not classified	1	0.58
Cytogenetic risk category	Favorable	35	20.47
	Intermediate	100	58.48
	Poor	33	19.30
	Unknow	3	1.75
Immunophenotype	CD33+	124	82.1
	CD34+	99	65.6
	CD117+	134	88.7
Mutation	DNMT3A	18	12.6
	FLT3	45	30.6
	NPM1	33	22.0
	RAS	8	5.3
	IDH1	26	17.2

### The Constructed Prognostic Model and Survival Analysis

**Figure 2:**
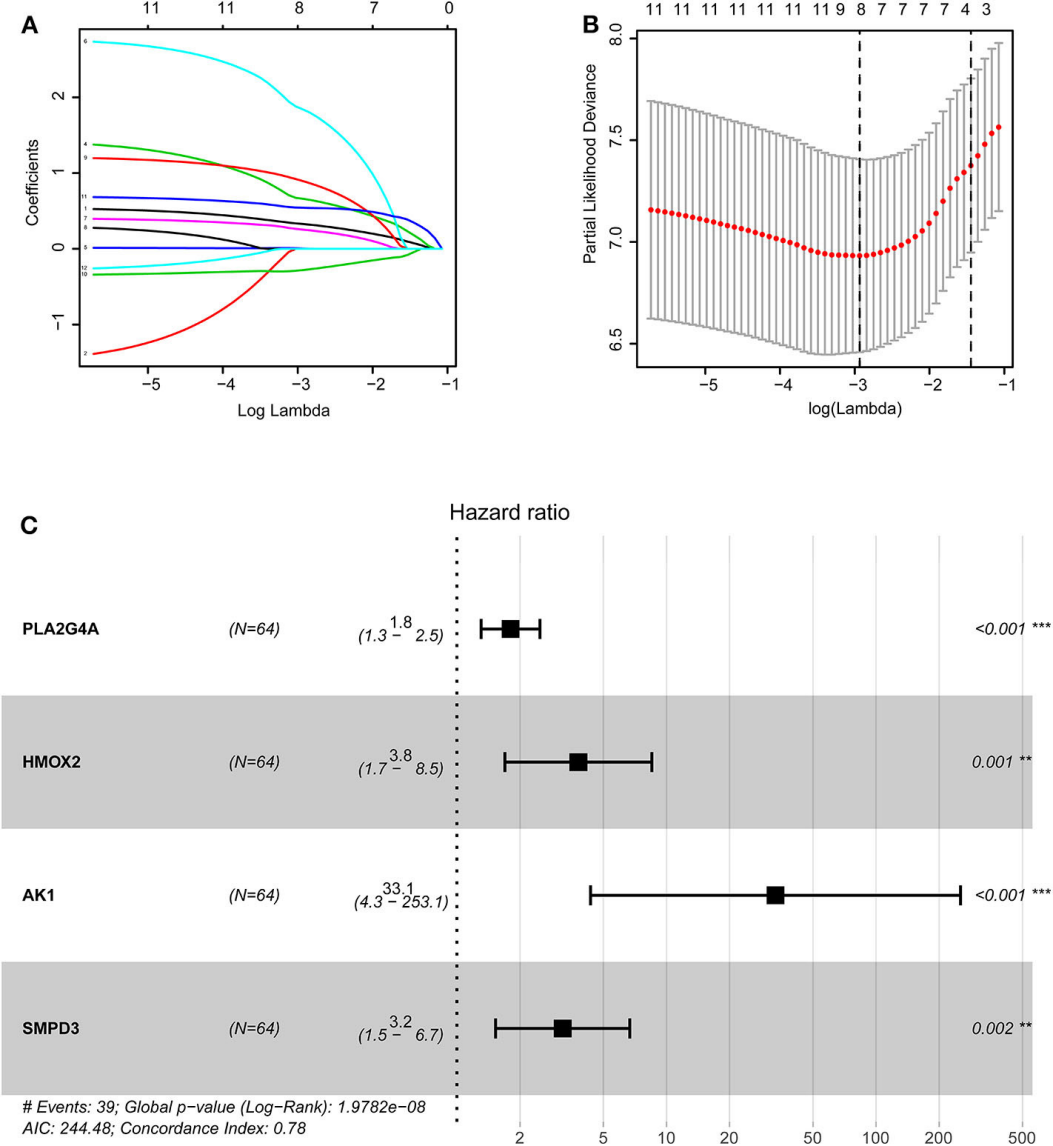
Construction of the prognostic model based on LASSO algorithm. **(A,B)** Figure **(B)** shows the Log Lambda value corresponding to the minimum cross-validation error point. And the metabolic genes with non-zero coefficient corresponding to the same Log Lambda value were selected in the **(A)** for subsequent model construction. **(C)** The forest map visually shows HR values and 95% confidence intervals for all model genes. HR <1 indicates that this gene is a low-risk gene; otherwise, it is a high-risk gene. *P* < 0.05 indicates that this gene is an independent prognostic factor.

**Figure 3:**
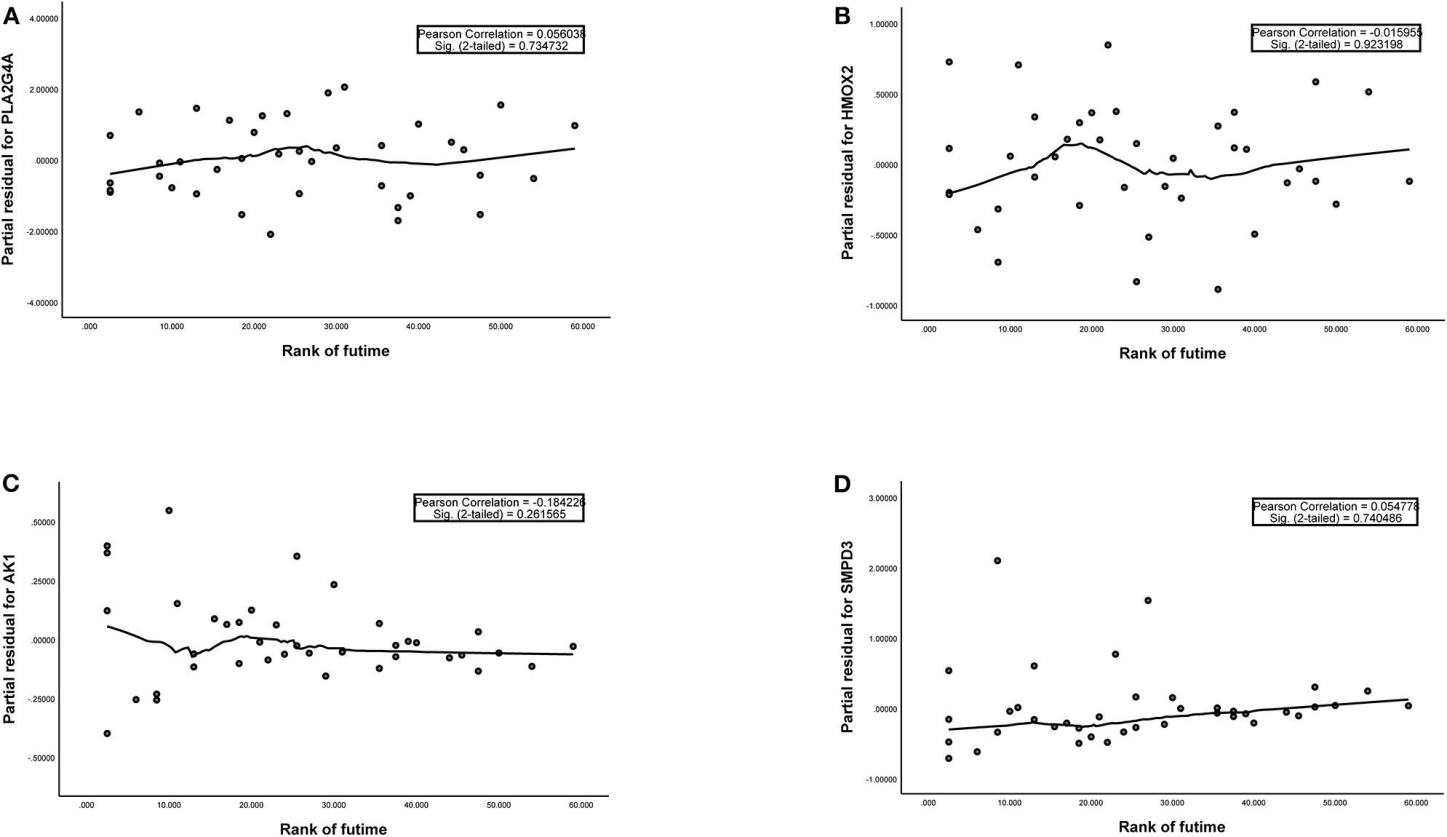
**(A–D)** The plots of the Schoenfeld Residuals against the transformed time for model genes. According to the variation trend of the smooth curve and statistical analysis, it can be determined that there is no correlation between the partial residuals of four model genes and time rank (*P* > 0.05), suggesting that all model genes meet the assumptions of proportional hazards and suitable for Cox regression analysis.

The training group (Supplementary File 3), the TCGA internal validation group (Supplementary File 4) and the GEO external validation groups (Supplementary Files 5, 6) were generated from one-time random grouping. After univariate COX analysis (*P* < 0.01) for the training group (Supplementary File 7), we put the 12 survival-related metabolic genes obtained into the LASSO algorithm (Figures 2A,B). After the stepwise multivariate Cox regression analysis, four metabolic genes realized the prognostic model construction finally. The final model formula is that each patient's riskScore = 0.588^*^PLA2G4A + 1.334^*^HMOX2 + 3.500^*^AK1 + 1.162^*^SMPD3 (Figure 2C, Table 2). All model genes are high-risk genes and independent prognostic factors, and also meet the assumptions of proportional hazards (Figure 3).

**Table 2:** Multivariate COX regression analysis results of model genes.

**Id**	**Coef**	**HR**	**HR.95L**	**HR.95H**	**P value**
PLA2G4A	0.588468436	1.801227607	1.30398577	2.488079983	0.000356501
HMOX2	1.333908151	3.795849187	1.692436314	8.513449476	0.001209189
AK1	3.500312611	33.12580585	4.336098654	253.0659703	0.000740836
SMPD3	1.162351687	3.197443829	1.529613372	6.683811234	0.00200342

**Figure 4:**
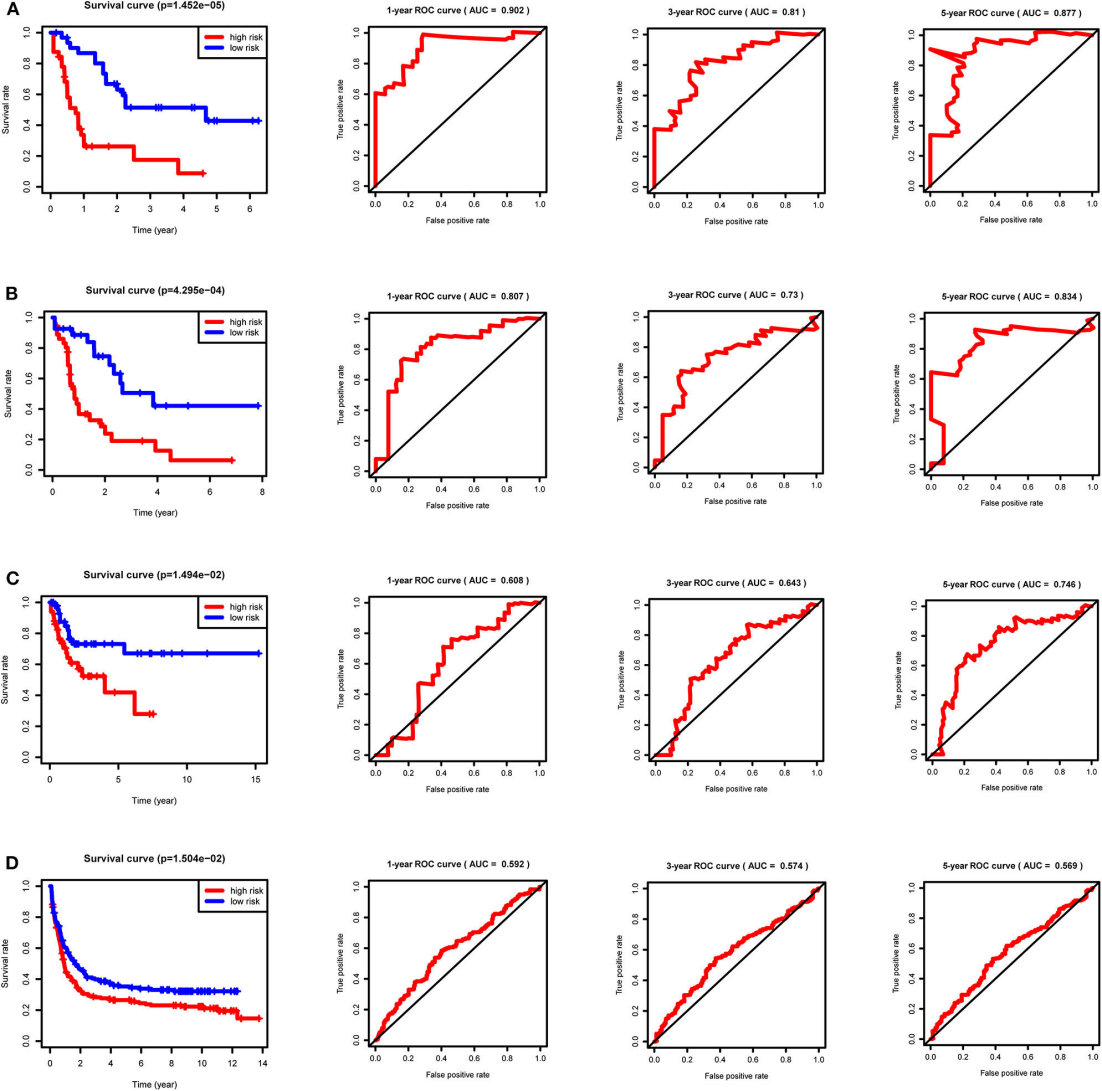
Survival curves and ROC curves for the training, internal validation and external validation groups. For the survival curves, survival rates decrease over time in both the high and low risk groups. *P* < 0.05 indicates survival differences between the high and low risk groups. For ROC curves, AUC > 0.9 suggests that the accuracy of the model is very high; The AUC value is between 0.7 and 0.9, indicating that the model has a certain accuracy. The AUC value is between 0.5 and 0.7, showing the accuracy of the model is general. **(A)** Training group. **(B)** Internal validation group. **(C)** External validation group GSE71014. **(D)** External validation group GSE37642.

**Figure 5:**
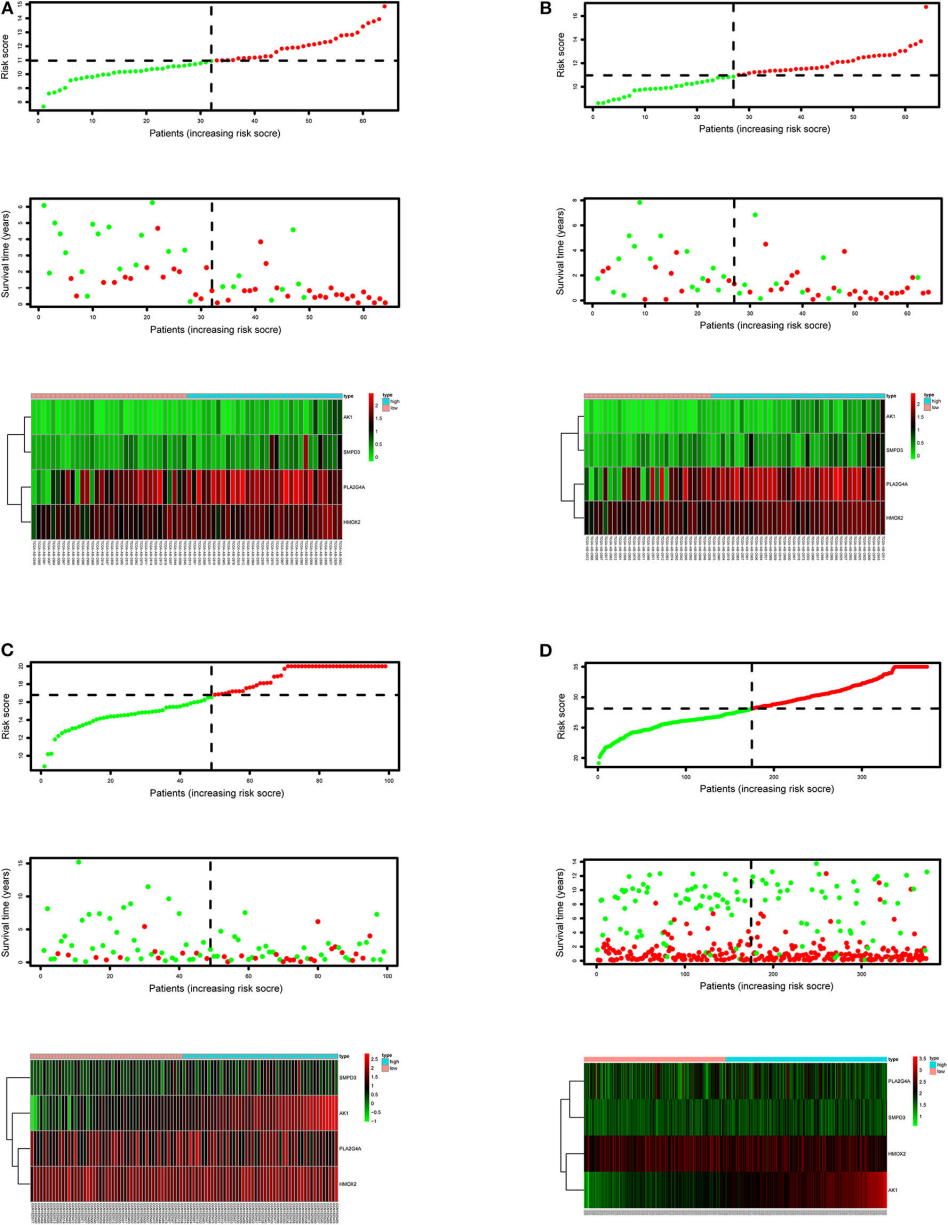
Risk score curves, survival status figures and risk heatmaps for the training, internal validation and external validation groups. The upper part of each subfigure represents the risk score curve (low risk patients are represented by green dots, high risk patients by red dots, and dash lines correspond to the median riskScore of training group). The middle section represents the distribution of survival status and survival time of patients ranked by riskScore (more green dots on the left for low-risk patients, and more red dots on the right for high-risk patients. From left to right, with the increase of the riskScore, more and more patients died, indicating that the riskScore is related to survival). The bottom heatmap displays the expressing pattern of the metabolic genes (the color transition from green to red indicates that the expressing level of the corresponding metabolic gene increases from low to high). **(A)** Training group. **(B)** Internal validation group. **(C)** External validation group GSE71014. **(D)** External validation group GSE37642.

**Figure 6:**
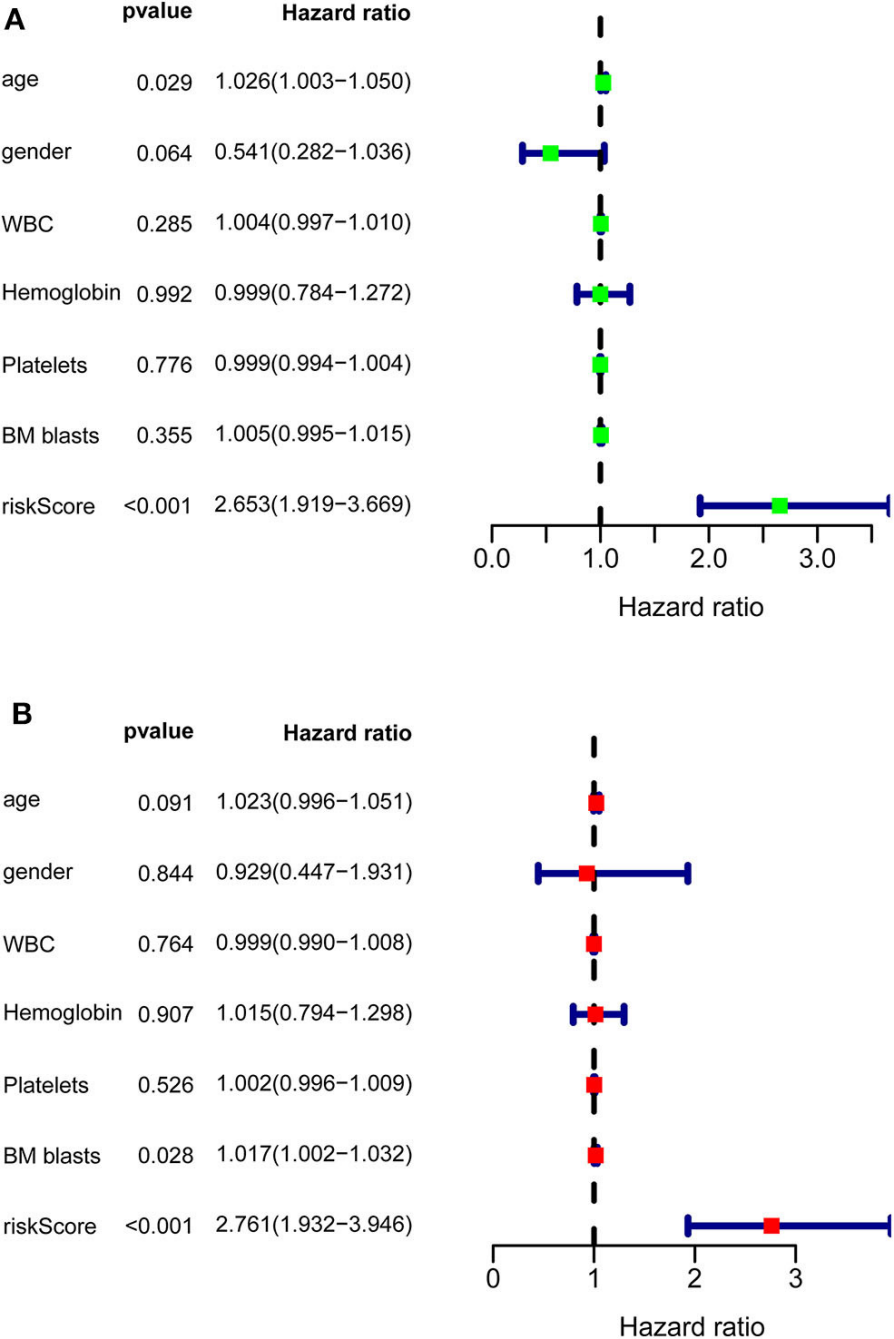
Univariate and multivariate prognostic analyses of clinical parameters and riskScore in TCGA-AML. **(A)** Univariate prognostic analyses. For a clinical parameter or riskScore, if the *P* < 0.05, it is related to survival; if HR >1, it is a high-risk factor. **(B)** Multivariate prognostic analyses. For the results of univariate and multivariate independent prognostic analysis, if the riskScore *P*-value of both is < 0.05, it indicates that riskScore is independent of other clinical parameters and can be used as an independent prognostic factor in clinical practice.

After calculating all patients' riskScores, we divided the patients in the training and validation groups into the high and low risk groups (Supplementary Files 8–11) for subsequent survival analysis. For the training group, the internal validation group and the external validation groups, the survival curves between the high and low risk groups have significant differences, and the survival rate of the low risk group is significantly higher than that of the high risk group. According to the AUC values of ROC curves, the accuracy of the prognostic model constructed by us is very high (Figure 4). Similarly, the risk curves make a good distinction between the high and low risk groups on the whole. For the upper risk score curves, the patient's riskScore increases from left to right. The middle survival status figures present the survival time decline and the mortality enhancement as the patients' riskScores increase. In the bottom risk heatmaps, each model gene expression increases as the patient's riskScore increases, indicating that all model genes are high-risk (Figure 5). Finally, univariate and multivariate prognostic analyses (*P* < 0.05) prove that the riskScore obtained from the model is an independent prognostic factor (Figure 6).

### ssGSEA Analysis and Mutation Data Visualization

**Figure 7:**
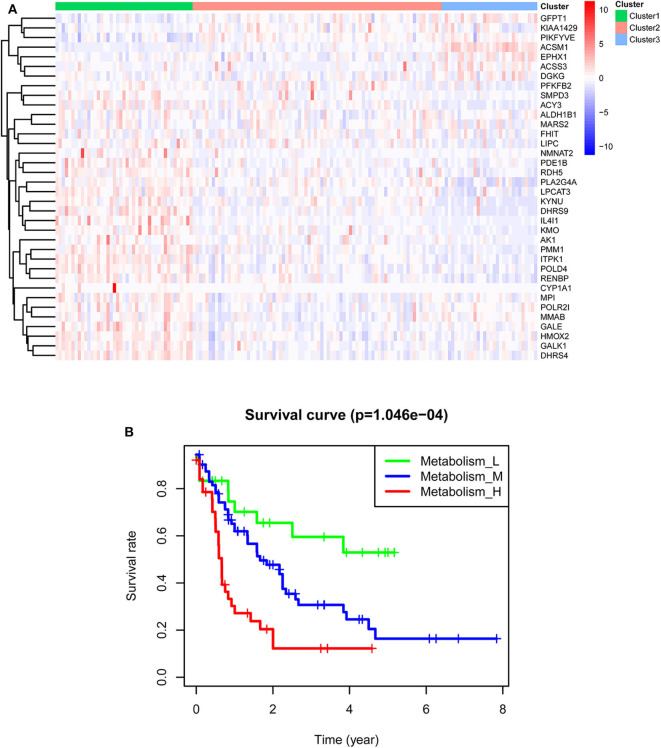
Clustering results of TCGA-AML samples. **(A)** Clustering heatmap. In the main body of the figure, red color represents the high expression of metabolic genes, and blue color represents the low expression of metabolic genes. Cluster1 is mainly red, that is, the metabolic genes are highly expressed, so it is a high metabolism group. Cluster2 has both red and blue colors, so it is a medium metabolism group. Cluster3 is mainly blue, that is, the metabolic genes are low expressed, so it is a low metabolism group. **(B)** Survival curve. *P* < 0.05 indicates that there is a difference in survival among the three groups.

**Figure 8:**
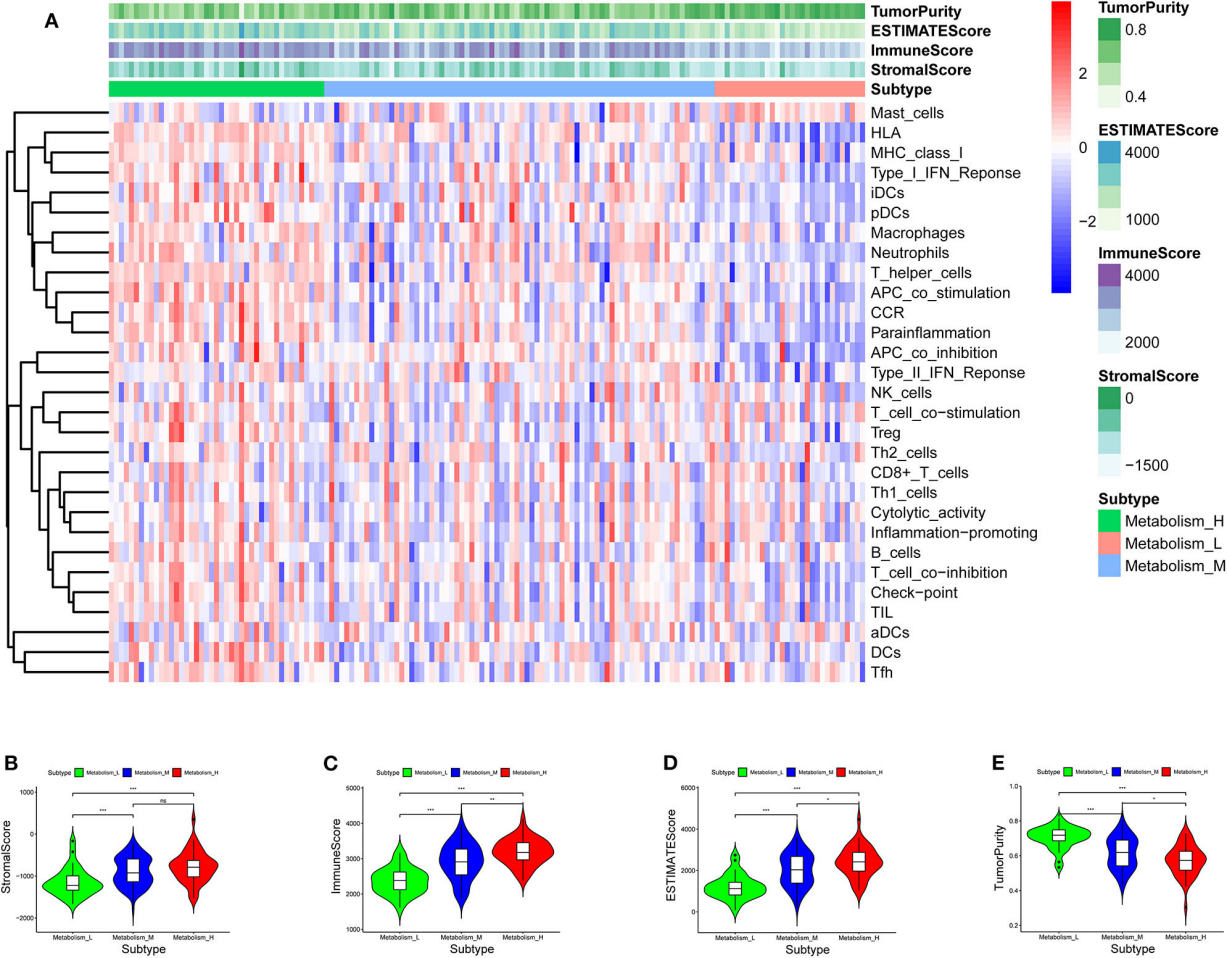
Heatmap and violin plot of tumor microenvironment. **(A)** Heatmap. The x-coordinate represents the sample names, and the y-coordinate represents the immune genesets. The upper part is the tumor microenvironment score, in which StromalScore is the stromal cell score (The color is lighter from left to right, the lower the score, the lower the stromal cell content. That is, with the decrease of metabolic activity, the content of stromal cells decreases), and ImmuneScore is the immune cell score (The color is lighter from left to right, the lower the score, the lower the immune cell content. That is, with the decrease of metabolic activity, the content of immune cells decreases), and ESTIMATEScore is the combined score of stromal cells and immune cells (The color is lighter from left to right, the lower the score, the lower the total content of stromal cells and immune cells. That is, with the decrease of metabolic activity, the total content of stromal cells and immune cells decreases). The higher ESTIMATEScore is, the lower TumorPurity is (The color is darker from left to right, the higher the score, the higher the tumor purity. That is, with the decrease of metabolic activity, the tumor purity increases). **(B–E)** Violin plot. With the increase of metabolic activity, the change trend of various tumor microenvironmental parameters can be seen. *P* < 0.001, *** *P* < 0.01, ** *P* < 0.05, * *P* >0.05, ns.

**Figure 9:**
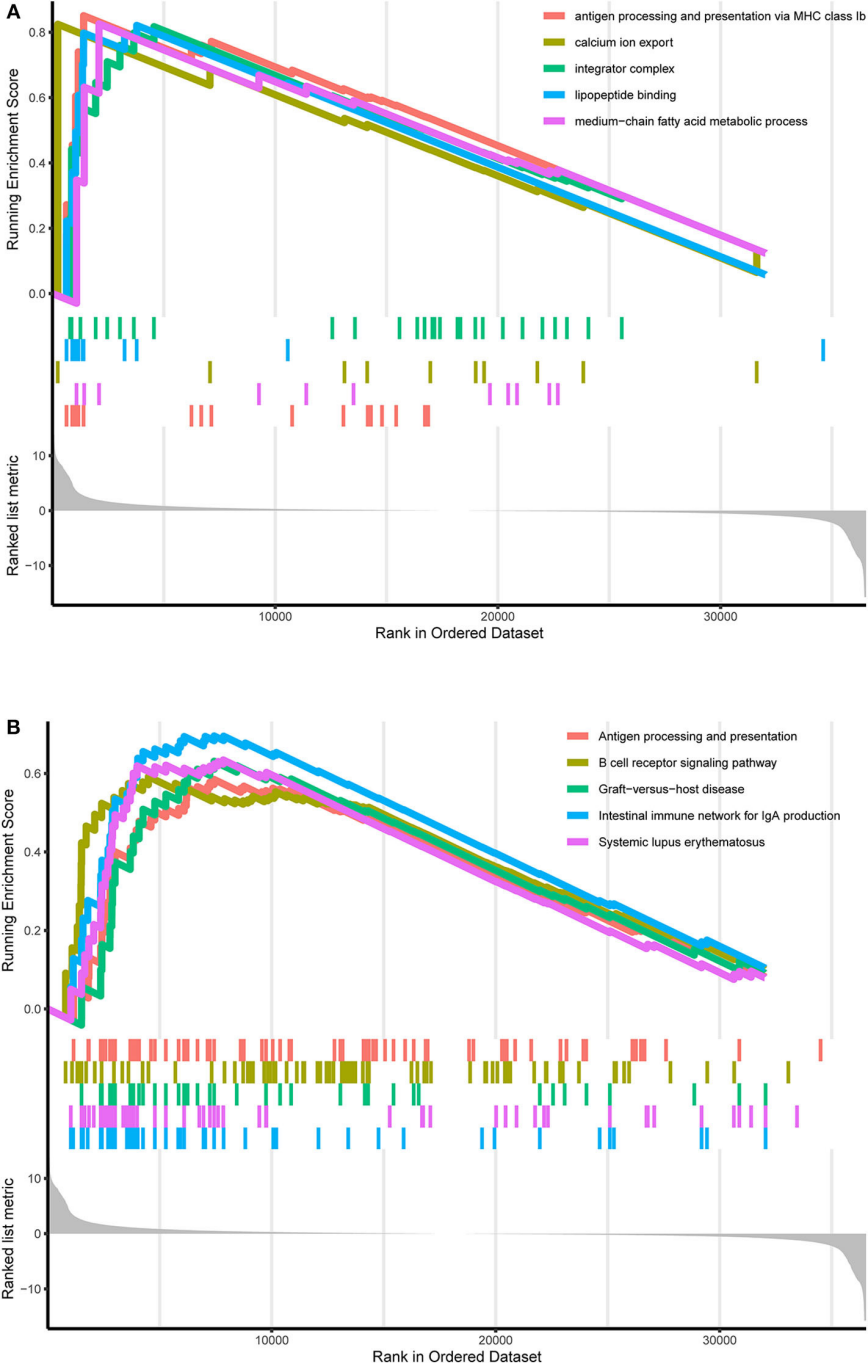
GO and KEGG enrichment results. **(A)** GO enrichment result. At the bottom, the genes are ranked by their logFC values (the closer to the left, the higher the logFC value; the closer to the right, the smaller the logFC value). In the middle section, each color represents the gene on the corresponding GO term. In the upper section, the highest score of each GO term is the GO enrichment score. These GO terms are the top 5 GO terms with the most significant enrichment in the high metabolism group. **(B)** KEGG enrichment result. At the bottom, the genes are ranked by their logFC values (the closer to the left, the higher the logFC value; the closer to the right, the smaller the logFC value). In the middle section, each color represents the gene on the corresponding KEGG pathway. In the upper section, the highest score of each KEGG pathway is the KEGG enrichment score. These KEGG pathways are the top 5 KEGG pathways with the most significant enrichment in the high metabolism group.

TCGA-AML samples were clustered to produce high, medium and low metabolism groups (Figure 7A, Supplementary File 12). The survival analysis reveals that there are significant differences among the three metabolism groups, and the lower the metabolic activity is, the longer the survival time is (Figure 7B). By analyzing the correlation between metabolism groups and tumor microenvironment, we plotted the heatmap (Figure 8A) and the violin plots (Figures 8B–E) of the tumor microenvironment. In summary, metabolic activity is positively correlated with StromalScore, ImmuneScore, and ESTIMATEScore, and negatively correlated with TumorPurity. GSEA enrichment analysis was carried out between high and low metabolism groups, obtaining the top 5 GO terms (Figure 9A) and KEGG pathways (Figure 9B) with most significant enrichment in high metabolism group (*P* < 0.05). These GO terms and KEGG pathways are primarily associated with immunity and metabolism.

**Figure 10:**
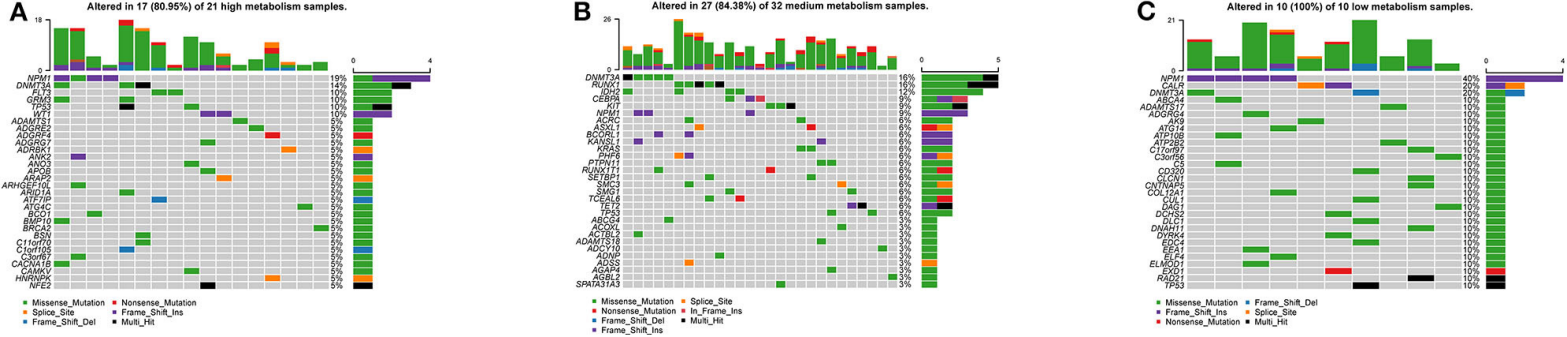
Waterfall plots, visualizing the top 30 mutation genes. The ordinate is the gene name, and the abscissa is the sample name. If the gene is not mutated in the sample, it is shown in gray. If the gene mutates in the sample, it is shown in other colors (different colors represent different mutation types). On the right, the number of samples in which the gene is mutated is counted. At the top, the number of mutations in the sample is counted. **(A)** High metabolism group. **(B)** Medium metabolism group. **(C)** Low metabolism group.

By visualizing the TCGA-AML mutation data, the waterfall plots of the high, medium and low metabolism groups generated (Figures 10A–C). It can be seen that there is no significant correlation between metabolic activity and total mutation frequency. The mutation frequency of NPM1 and DNMT3A are the highest among the three metabolism groups. Among them, NPM1 mutation is mainly frameshift insert mutation, and DNMT3A mutation is mostly missense mutation.

### Mutation Status and Multiple Validations of Model Genes

**Figure 11:**
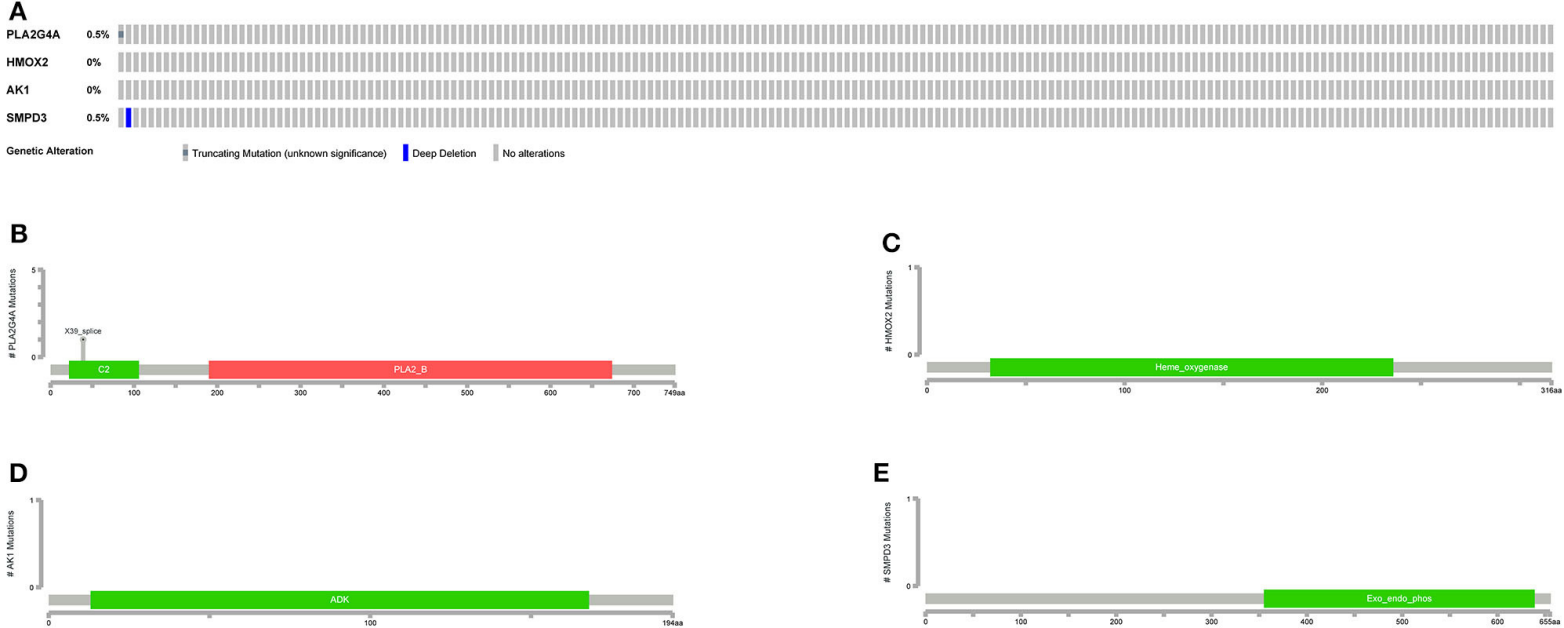
Mutations in model genes. **(A)** The general picture of genetic alteration (mutation and copy number variation). One rectangle represents a sample. The gray rectangle represents no alteration in the sample, and the other colors represent alterations in the sample (different colors represent different alteration types). On the left, the alteration frequency of each gene in all samples is counted. **(B–E)** Mutation patterns in gene domains. The x-coordinate represents the number of amino acid bases, and the y-coordinate represents the number of mutant samples. Different colors represent different domains.

The general picture of genetic alteration (Figure 11A) and the domain mutation diagrams (Figures 11B–E) of the model genes were downloaded from the cBioportal website. The mutation frequencies of these model genes are very low, and the mutations hardly affect the functional change of the structural domains.

**Figure 12:**
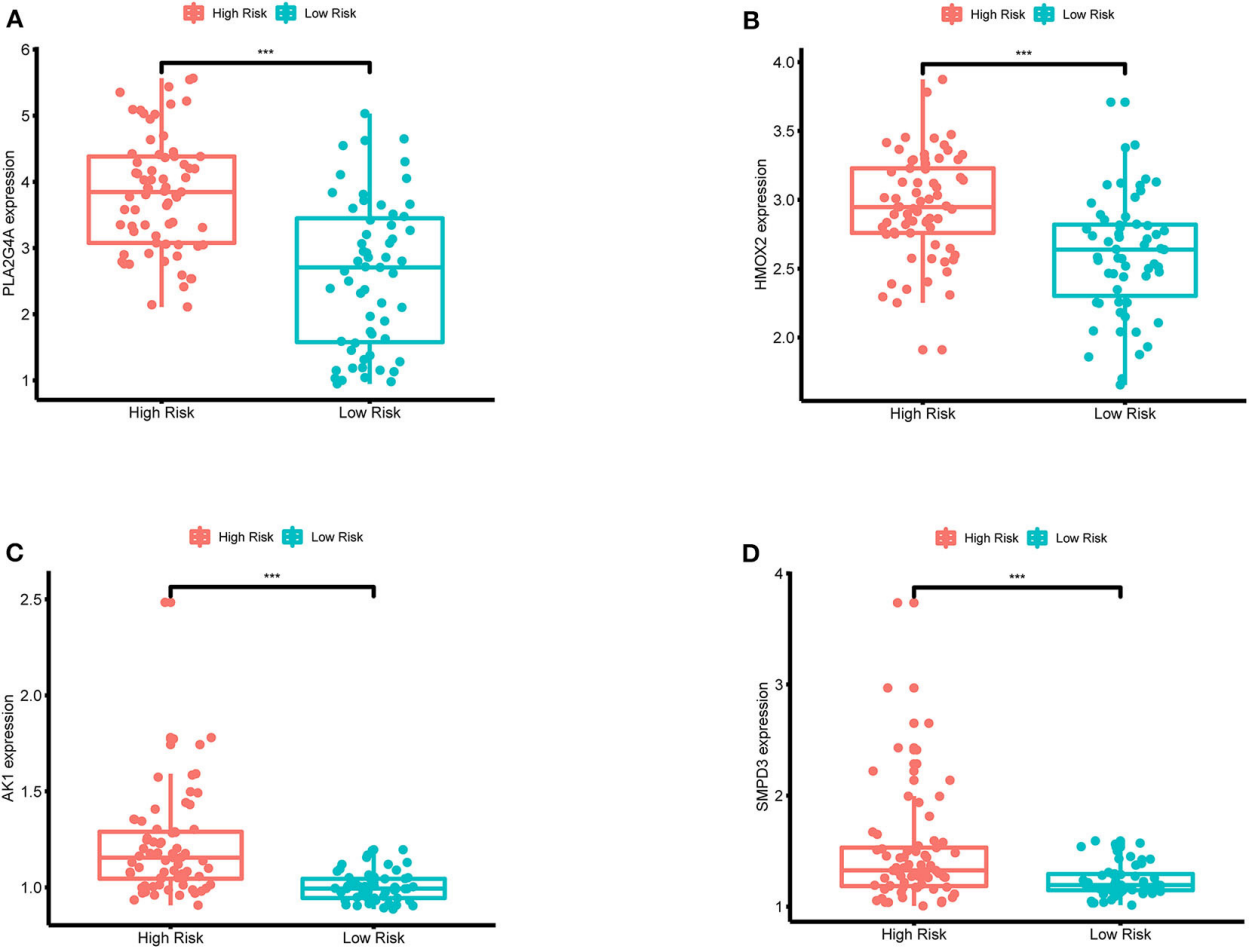
**(A–D)** Boxplots for the difference validation of model genes. The abscissa is the grouping type, and the ordinate is the expression quantity of the model genes. All model genes are highly expressed in the high-risk group (*P* < 0.001), suggesting that all model genes are high-risk genes. ***, *P* < 0.001.

In order to validate the expression differences of model genes between the high-risk group and the low-risk group, boxplots of model genes were drawn (Figure 12). All model genes are significantly highly expressed in the high-risk group, which to some extent supports the previous conclusion that all model genes are high-risk genes.

**Figure 13:**
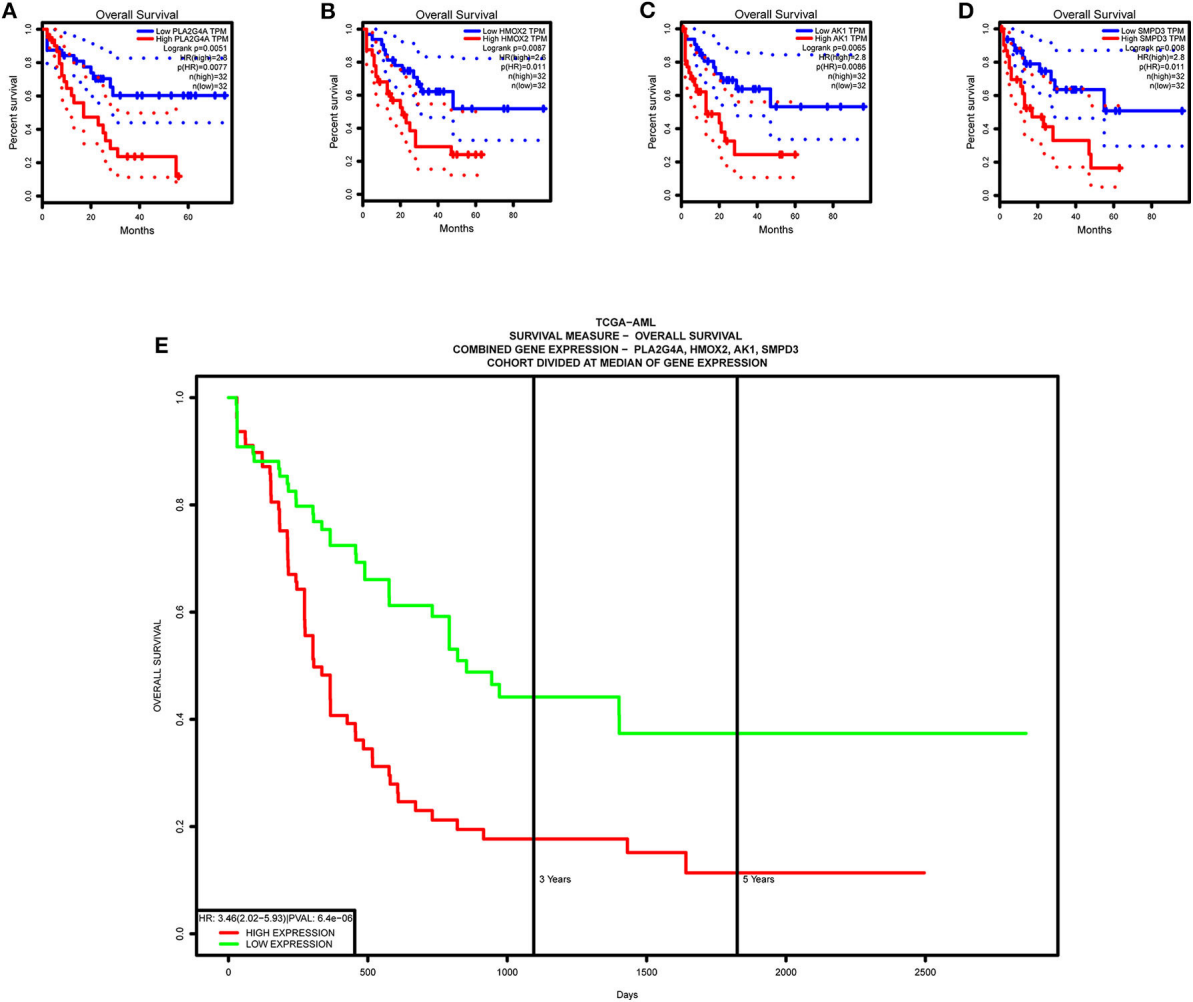
Survival validation of model genes. **(A–D)** Single-gene survival analysis, from the GEPIA website. **(E)** Multiple gene survival analysis, from the PROGgeneV2 online tool.

Single-gene survival analysis again validates that each model gene is a high-risk gene and has a significant impact on the prognosis of AML patients (Figures 13A–D). Furthermore, the multiple gene survival analysis combining all model genes successfully validates the accuracy and effectiveness of the prognostic model we constructed (Figure 13E).

## Discussion

The exploration of metabolic prognostic effects has broadened our horizon and our understanding of traditional transcriptome molecular biomarkers. In this study, we adopted a variety of bioinformatics methods to mine prognosis-related metabolic genes by integrating the metabolic characteristics and clinical information of AML patients, and constructed an accurate and efficient metabolic prognosis model with internal and external validations. Furthermore, the association between metabolism and immunity in AML was also explored, as well as the mutation situation. Finally, multiple validations of the model genes further improved the rigor of our study.

Current diagnosis and treatment of AML rely on histopathological diagnosis and clear classification. With the application of second-generation sequencing and other technologies, AML has more and more molecular targets, which has led to the rapid development of targeted drugs, such as some small molecular targeted drugs, immune-targeted drugs and all kinds of cutting-edge new drugs in clinical research, so the choice of treatment has become more diversified. Therefore, potential biomarkers can be mined and used to predict patient outcomes and develop new treatment strategies.

The next-generation sequencing technology developed in recent years employs the whole genome sequencing method, which brings great advantages to multi-group data mining. Prior to this, some studies performed metabolic analysis for some types of cancer and constructed prognostic signatures for cancer prognosis monitoring, including clear cell renal cell carcinoma (35), endometrial adenocarcinoma (36) and glioma (37). This computational bioinformatics analysis can open different perspectives on the clinical application and potential pathological mechanisms of metabolic biomarkers at the macro level. A number of previous studies have proposed transcriptome signatures associated with the prognosis of AML through bioinformatics analysis (38–40). Our study further explored metabolic biomarkers as prognostic predictors and broadened our understanding of the transcriptome clinical significance.

Multiple validations can basically determine that the metabolic model genes we have found are high-risk genes and independent prognostic factors. A recent paper shows that bioinformatic analysis identifies that PLA2G4A has physical interactions with several oncogenic proteins (such as RUVBL2, CAP1, STAT3, and MYCBP) in AML, resulting in multiple effects on the malignant phenotype of AML cells (41). Also, another recent paper reports that AK1 is an independent adverse prognostic factor for AML patients receiving chemotherapy, and patients with high AK1 expression may be recommended for early Allo-HSCT (42). Current studies on the roles of HMOX2 and SMPD3 in AML are largely blank, but they play important roles in other cancers (43, 44), so more preliminary single-gene bioinformatics analyses are needed. Based on previous studies in AML, PLA2G4A and AK1 may be new oncogenes that are more worthy of molecular functional experiments to explore further.

ssGSEA analysis reveals that overall metabolic activity is positively correlated with immune activity in AML. There are two undeniable trends. Firstly, cancer cells tend to have more vigorous metabolism in order to absorb more energy and proliferate malignantly. Secondly, specific antigens of cancer cells can be recognized by the immune system, and the activated immune cells can further play a killing role on the tumor. Based on the survival differences between the three metabolism groups, these two common senses may provide a crude explanation for the high metabolic activity associated with high immune activity in AML. However, the mysteries of the human body are always more complex and wonderful than we think. The crosstalk between different metabolic pathways will seriously affect the tumor microenvironment and eventually impair the immune cells' adaptability and effector function, restricting the success of immunotherapy (17). In tumors, metabolism and immunity do not have a strictly one-way cause-and-effect relationship; More precisely, they always cause and affect each other. In recent years, the most popular studies have always been related to the relationship between metabolic reprogramming and immune escape in tumors (45, 46), including glycolysis with general significance (47). Metabolic reprogramming associated with AML cells, including competition with substrates, large release of bioactive metabolites and overall microenvironmental metabolic remodeling conducive to the survival of immunoregulatory cell subgroups, has become an important mechanism of immune escape in AML and severely hinders the efficacy of immunotherapy (13). Nevertheless, these studies of metabolic changes at the micro level do not contradict our findings, as our conclusion is for the overall metabolic activity of AML at the macro level.

Similarly, GSEA enrichment results in the high metabolism group are mainly related to immunity and metabolism, which is also a supporting basis for the previous ssGSEA analysis results, in which immunity includes antigen processing and presentation, B cell receptor signaling pathway, intestinal immune network for IgA production and lipopeptide binding, presenting pervasive immune response processes; metabolism includes calcium ion export, integrator complex and medium–chain fatty acid metabolic process, also involving a wide range of metabolic processes. As the research object is the overall metabolic activity, GSEA enrichment results are not limited to a single type of biological process. Previous studies have shown that B-cell receptor signal transduction strictly regulates the growth and proliferation of B-cells, and activated B-cells respond to changes in energy and biosynthetic demands and conduct metabolic reprogramming to adapt to metabolic pressure in tumors (48). Another focus of AML research is the intestinal flora. Intestinal flora can keep people healthy by participating in the metabolic process and regulating the immune system, and the reconstruction of healthy intestinal microflora by adjusting the diet can improve the progression and prognosis of AML (49). Calcium signal transduction plays a key role in several biological processes, such as cell growth, differentiation, metabolism and death, and abnormal calcium signaling and loss of calcium homeostasis can lead to tumor proliferation, angiogenesis and other vital processes of cancer progression (50).

The results of mutation analysis indicate that the mutation frequencies of other genes, especially our model genes, are not high except for the well-known two genes, NPM1 and DNMT3A. Perhaps from the side, our model genes do not depend on structural and functional changes, that is, qualitative changes, to affect the prognosis of AML; they play high-risk roles in the prognosis of AML by the quantitative change. This point can be inferred from our previous discussion on model genes (41–44).

We believe that the data of public platforms we use are appropriately standardized, and multiple validations enhance the rigor of our research. In addition, our conclusion needs to be further validated by wet experiments to be more convincing.

## Conclusions

In conclusion, we adopted a variety of bioinformatics methods to establish an accurate and efficient prognostic model for AML patients, and carried out multiple validations. In addition, the relationship between metabolism and immunity and mutations in AML were explored. These findings provide fundamental insights into the molecular mechanisms and diagnostic markers of AML and contribute to the development of new genomic models for clinical cancer management.

## Data Availability Statement

All datasets generated for this study are included in the article/Supplementary Material.

## Author Contributions

YZ and SM conceived of and designed the study. YZ, SM, and MW performed the literature search, generated the figures and tables, and wrote the manuscript. WS and YH supervised the study and reviewed the manuscript. All authors read and approved the final manuscript.

## Conflict of Interest

The authors declare that the research was conducted in the absence of any commercial or financial relationships that could be construed as a potential conflict of interest.

## Abbreviations

AML: Acute myeloid leukemia
GTEx: Genotype-Tissue Expression
TCGA: The Cancer Genome Atlas
GEO: Gene Expression Omnibus
GSEA: Gene set enrichment analysis
ssGSEA: single-sample gene set enrichment analysis
AUC: Area under the curve
ROC: Receiver operating characteristic
HSC: Hematopoietic stem cell
TME: Tumor microenvironment
UCSC: University of California Santa Cruz
LASSO: Least absolute shrinkage and selection operator
GO: Gene ontology
KEGG: Kyoto encyclopedia of genes and genomes
GEPIA: Gene Expression Profiling Interactive Analysis.
